# Copolymerization of epoxides with cyclic anhydrides catalyzed by dinuclear cobalt complexes

**DOI:** 10.3762/bjoc.14.255

**Published:** 2018-11-05

**Authors:** Yo Hiranoi, Koji Nakano

**Affiliations:** 1 Department of Organic and Polymer Materials Chemistry, Tokyo University of Agriculture and Technology, 2-24-16 Naka-cho, Koganei, Tokyo 184-8588, Japan

**Keywords:** cobalt, copolymerization, cyclic anhydrides, epoxides, polyesters

## Abstract

The alternating copolymerization of epoxides with cyclic anhydrides (CAs) is a highly diverse synthetic method for polyesters as the polymers’ architectures and properties can be easily controlled depending on the combination of two monomers. Thus, a variety of catalyst designs has been reported to prepare the desired copolymers efficiently. We herein report dinuclear cobalt–salen complexes with a benzene ring as a linker and their activities in copolymerization reactions. The dinuclear cobalt complexes showed a higher catalytic activity for the copolymerization of propylene oxide with phthalic anhydride than the corresponding mononuclear cobalt–salen complex and achieved one of the highest turnover frequencies ever reported. A variety of epoxides and CAs were also found to be copolymerized successfully by the dinuclear cobalt complex with a high catalytic activity.

## Introduction

Aliphatic polyesters have received significant attention owing to their good biocompatibility and biodegradability [1–4]. These attractive features allow them to be applied in medical and ecological materials as well as in commodity materials. The conventional way of synthesizing polyesters is the step-growth polymerization of diacids (or diacid derivatives) with diols. The ready availability of structurally diverse diacids and diols provides access to a wide range of polyesters. In this method, an extremely high conversion of the carboxy and hydroxy groups should be achieved for synthesizing high molecular weight polyesters. However, there are some burdensome requisites, such as a precise stoichiometric balance between the carboxy and hydroxy groups and an efficient removal of small molecule byproducts, for the high conversion. Another conventional method for polyester synthesis is the chain-growth ring-opening polymerization (ROP) of lactones and cyclic diesters [5–10]. In contrast to the step-growth polymerization, the ROP does not give any small molecule byproducts and proceeds under mild conditions. In addition, high molecular weight polyesters with narrow polydispersity can be prepared even at a low monomer conversion. A variety of lactones and cyclic diesters, such as ε-caprolactone, β-propiolactone, lactic acid (LA), and glycolide have been used for the ROP. However, the employable monomers are rather limited, which restricts the range of polymer properties.

In view of the aforementioned, the alternating copolymerization of epoxides with cyclic anhydrides (CAs) is a promising alternative for polyester synthesis [11–12]. A broad range of epoxides and CAs are readily available and can be copolymerized through this method. Therefore, the polymer architectures and properties can be easily controlled depending on the combination of the two monomers used. This copolymerization method was first reported in 1960 where a tertiary amine was used as a catalyst [13]. Since then, a range of polymerization catalysts including alkyl metals and inorganic salts have been reported. However, the development of the epoxide/CA copolymerization was constricted until recently because of low catalytic activity and poor control over the main chain sequence (formation of ether linkages through consecutive epoxide enchainment) and molecular weight. In 2007, Coates and co-workers reported that β-diiminate zinc complexes exhibited a high catalytic activity for the epoxide/CA copolymerization [14]. The resulting polyesters were found to possess completely alternating structures with high molecular weight and relatively narrow polydispersity. Following this report, a range of highly active and/or selective catalysts has been developed based on well-defined metal complexes such as metalloporphyrins and metal–salen complexes [15–21]. In parallel to the development of catalysts, new polyester materials also were prepared by employing unprecedented monomers or by the combination with other polymerization methods [22–26].

**Figure 1:**
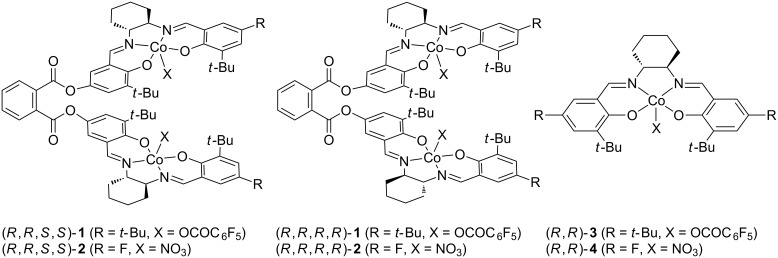
Structures of cobalt–salen complexes **1**–**4**.

Cooperative dinuclear metal catalysts have been considered as a promising design for high activity and/or selectivity in organic transformations including polymerizations [27–30]. This was found to be true for the epoxide/CA copolymerization. In 2013, Lu and co-workers reported that the dinuclear chromium–salan complex showed a much higher catalytic activity than the corresponding mononuclear chromium–salan complex in the copolymerization of epoxides with maleic anhydride (MA) [31]. Following this report, some dinuclear metal complexes have been reported to demonstrate high and/or unique catalytic performances in the epoxide/CA copolymerization [23,32–39]. Recently, we have reported the dinuclear cobalt–salen complexes as catalysts for the copolymerization of epoxides with carbon dioxide (CO_2_), affording superior catalytic activity compared to the corresponding mononuclear cobalt–salen complexes [40]. During the course of our study, the dinuclear cobalt–salen complex (*R*,*R*,*S*,*S*)-**1** was found to exhibit a high catalytic activity for the alternating copolymerization of propylene oxide (PO) with phthalic anhydride (PA, Figure 1). The observed catalytic activity was much higher than that achieved by using the mononuclear cobalt–salen complex. Although mononuclear cobalt–salen complexes are known as one of the most active catalysts for the epoxide/CA copolymerization [15], there has been no report on the catalyst design based on dinuclear cobalt–salen complexes. This context prompted us to explore the catalyst performance of the dinuclear cobalt–salen complexes. Herein we report our further investigation on the epoxide/CA copolymerization by using dinuclear cobalt–salen complexes.

## Results and Discussion

### Synthesis of dinuclear cobalt–salen complexes

In our previous preliminary investigation, we used the heterochiral dinuclear cobalt–salen complex (*R*,*R*,*S*,*S*)-**1** with *tert*-butyl groups at the 5-positions of the salicylidene moieties and a pentafluorobenzoate group as an axial ligand [40]. Recently, the substituents at the 5-positions and the axial ligand of mononuclear cobalt–salen complexes were proven to have a great impact on the catalytic activity [15]. The electron-withdrawing fluoro group at the 5-positions and a nitrate axial ligand gave the most active catalyst for the PO/MA copolymerization. Accordingly, we designed the heterochiral dinuclear cobalt–salen complex (*R*,*R*,*S*,*S*)-**2** as well as the homochiral complex (*R*,*R*,*R*,*R*)-**2** with fluoro groups at the 5-positions and nitrate axial ligands.

**Scheme 1:**
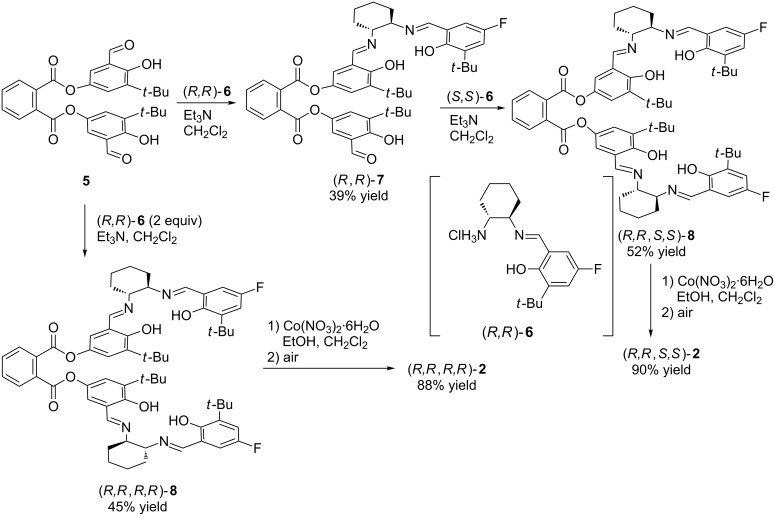
Synthesis of dinuclear cobalt–salen complexes (*R*,*R*,*S*,*S*)-**2** and (*R*,*R*,*R*,*R*)-**2**.

The heterochiral bis(salen) ligand precursor (*R*,*R*,*S*,*S*)-**8** was synthesized according to the procedure we have reported previously (Scheme 1) [40]. First, the reaction of bis(salicylaldehyde) **5** with (*R*,*R*)-half-salen **6**, which was prepared from (*R*,*R*)-1,2-cyclohexanediamine monohydrochloride and 3-*tert*-butyl-5-fluoro-2-hydroxybenzaldehyde, gave monosalen (*R*,*R*)-**7** in 39% yield. Then, the obtained mono(salen) (*R*,*R*)-**7** was converted into the heterochiral bis(salen) ligand precursor (*R*,*R*,*S*,*S*)-**8** in 52% yield through the condensation with (*S*,*S*)-half-salen **6**. In addition, the homochiral bis(salen) ligand precursor (*R*,*R*,*R*,*R*)-**8** was prepared by the reaction of bis(salicylaldehyde) **5** with two equivalents of (*R*,*R*)-half-salen **6** in 45% yield. Both, (*R*,*R*,*S*,*S*)-**8** and (*R*,*R*,*R*,*R*)-**8** were then treated with cobalt(II) nitrate and the following oxidation under air afforded the corresponding dinuclear cobalt–salen complexes (*R*,*R*,*S*,*S*)-**2** and (*R*,*R*,*R*,*R*)-**2**, respectively.

### Copolymerization of propylene oxide with phthalic anhydride

The catalysts’ performances were evaluated through the copolymerization of PO with PA (Table 1). For a convenient comparison of the different catalytic systems, the catalytic activity is expressed in terms of turnover frequency [TOF, (mol of PA incorporated in the copolymer)·(mol of Co center)^−1^·h^−1^]. First, the copolymerization was conducted in the presence of the homochiral dinuclear cobalt–salen complex (*R*,*R*,*R*,*R*)-**1** and [Ph_3_P=N=PPh_3_][OCOC_6_F_5_] ([PPN][OCOC_6_F_5_]) as the co-catalyst at 30 °C for 1 h (Table 1, entry 1, [PO]/[PA]/[Co] (= 2[(*R*,*R*,*R*,*R*)-**1**])**/**[co-catalyst] = 4,000:400:1:1). Since the solubility of PA in PO at 30 °C is limited, a large excess of PO over PA was used to maintain a homogeneous system. Under these conditions, a high catalytic activity with a TOF of 299 h^−1^ was accomplished. A decrease in the amount of co-catalyst resulted in a lower TOF (Table 1, entry 2) and the copolymerization did not proceed at all in the absence of co-catalyst (Table 1, entry 3). Thus, one equivalent of a co-catalyst is necessary for a high catalytic activity. A remarkably high TOF of 908 h^−1^ was achieved at the higher copolymerization temperature of 60 °C (Table 1, entry 4). This is one of the highest TOF values ever reported for the metal-catalyzed PO/PA copolymerization [41].

**Table 1:** Copolymerization of propylene oxide (PO) with phthalic anhydride (PA) using cobalt–salen complexes.^a^

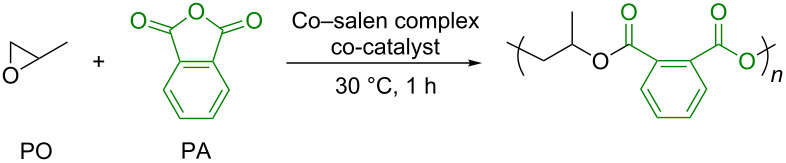					

entry	Co–salen complex	co-catalyst	TOF^b^ (h^−1^)	*M*_n_^c^	*M*_w_/*M*_n_^c^

1	(*R*,*R*,*R*,*R*)-**1**	[PPN][OCOC_6_F_5_]	299	15,500	1.13
2^d^	(*R*,*R*,*R*,*R*)-**1**	[PPN][OCOC_6_F_5_]	132	7,800	1.13
3	(*R*,*R*,*R*,*R*)-**1**		0	–^e^	–^e^
4^f^	(*R*,*R*,*R*,*R*)-**1**	[PPN][OCOC_6_F_5_]	908	12,000	1.14
5^g^	(*R*,*R*,*R*,*R*)-**1**	[PPN][OCOC_6_F_5_]	149	7,700	1.18
6	(*R*,*R*,*S*,*S*)-**1**	[PPN][OCOC_6_F_5_]	237	13,700	1.14
7	(*R*,*R*)-**3**	[PPN][OCOC_6_F_5_]	203	11,400	1.14
8^g^	(*R*,*R*)-**3**	[PPN][OCOC_6_F_5_]	50	–^e^	–^e^
9	*rac*-**3**	[PPN][OCOC_6_F_5_]	207	13,600	1.16
10	(*R*,*R*,*R*,*R*)-**2**	[PPN][NO_3_]	33	–^e^	–^e^
11	(*R*,*R*,*R*,*R*)-**2**	[PPN][OCOC_6_F_5_]	31	–^e^	–^e^
12	(*R*,*R*,*S*,*S*)-**2**	[PPN][NO_3_]	12	–^e^	–^e^
13	*rac*-**4**	[PPN][NO_3_]	107	5,400	1.25

^a^Copolymerization conditions: PO (20 mmol), PA (2.0 mmol), cobalt complex, and [PPN][OCOC_6_F_5_] as co-catalyst at 30 °C for 1 h. [PO]/[PA]/[Co]/[co-catalyst] = 4,000:400:1:1. ^b^Turnover frequency (TOF) = (mol of PA incorporated in the copolymer)·(mol of Co center)^−1^·h^−1^ calculated based on the ^1^H NMR spectrum of the polymerization mixture using phenanthrene as an internal standard. ^c^Estimated by size-exclusion-chromatography analysis using a polystyrene standard. ^d^[PO]/[PA]/[Co]/[co-catalyst] = 4,000:400:1:0.5. ^e^Not determined because of no or low conversion of PA. ^f^60 °C for 15 min. ^g^[PO]/[PA]/[Co]/[co-catalyst] = 4,000:400:0.25:0.25.

**Figure 2:**
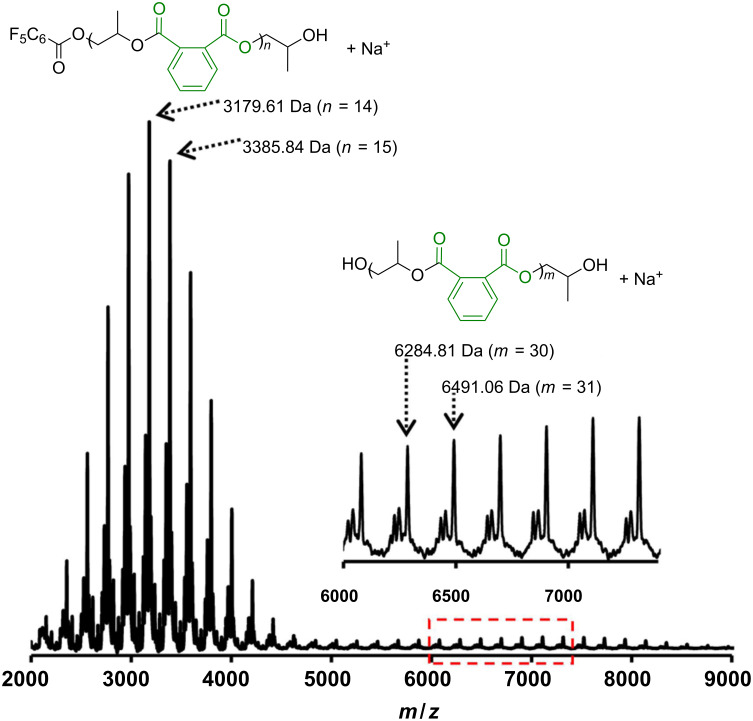
MALDI–TOF mass spectrum of the PO/PA copolymer. The low molecular weight copolymer for MS analysis was prepared by using (*R*,*R*,*R*,*R*)-**1** at 30 °C for 15 min.

As there were no detectable signal for ether linkages in the ^1^H NMR spectra of the obtained copolymers, the resulting copolymers possess a completely alternating structure. The regioselectivity in the ring-opening of PO was estimated by analyzing the stereochemistry of the copolymer prepared from enantiomerically pure (*S*)-PO. Because a ring opening at the methine carbon results in both a regioerror and the inversion of stereochemistry at the stereocenter, the enantiomeric excess (ee) of the repeating unit in the copolymer should reflect the regioregularity [15]. The ee of propylene glycol, which was obtained after hydrolysis of the copolymer obtained with (*S*)-PO and PA, was found to be 88%, indicating a high level of regioregularity. Molecular weight distributions are relatively narrow, while a bimodal distribution was observed in the SEC traces. The peak molecular weight of the higher molecular weight portion was twice as large as that of the lower molecular weight portion. Accordingly, trace amounts of diacid in PA and/or water contaminants would work as bifunctional chain-transfer agents and gave twice the molecular weight of the copolymer initiated by monofunctional pentafluorobenzoate from (*R*,*R*,*S*,*S*)-**1** and [PPN][OCOC_6_F_5_] [19]. The formation of the alternating copolymers initiated by monofunctional pentafluorobenzoate and the bifunctional chain-transfer agents was confirmed by matrix-assisted laser desorption/ionization time-of-flight mass spectrometry (MALDI–TOF MS, Figure 2). Several series of signals with a regular interval of 206.1 (repeating unit) were observed in the lower mass range. The *m*/*z* value of each signal of the major distribution corresponds with [166.0 (C_6_F_5_COO, initiating group) + 206.1*n* (repeating unit) + 59.1 (CH_2_CHMeOH, terminal group) + 23.0 (Na^+^ ion)]. Thus, the expected α-C_6_F_5_COO,ω-OH-terminated copolymer was obtained as a main product. As a minor distribution, the α-C_6_F_5_COO,ω-COOH-terminated copolymer was observed, while the cyclic polyester via intramolecular transesterification was not detected (Supporting Information File 1, Figure S14). In a higher mass range, a series of signals for the copolymer with hydroxy groups at both chain ends (the α-OH,ω-OH-terminated copolymer) was observed as the major distribution along with the α-OH,ω-COOH-terminated copolymer as the minor distribution.

Next, we investigated the effect of linking two cobalt–salen complexes on the catalytic activity. The heterochiral complex (*R*,*R*,*S*,*S*)-**1** demonstrated a much lower TOF of 237 h^−1^ than the homochiral one (*R*,*R*,*R*,*R*)-**1** (Table 1, entry 6). Thereby, a combination of the same absolute configuration of two salen moieties was found to be favorable for a high catalytic activity. Such dependence of the catalytic activity on the absolute configuration contrasts with our previous observation in the epoxide/CO_2_ copolymerization where the heterochiral (*R*,*R*,*S*,*S*)-**1** showed a much higher catalytic activity than the corresponding homochiral complex (*R*,*R*,*R*,*R*)-**1** [40]. Both mononuclear complexes (*R*,*R*)-**3** and *rac*-**3** demonstrated similar and relatively high TOFs of 203 and 207 h^−1^, respectively (Table 1 entries 7 and 9). However, these TOFs are lower than those obtained with the dinuclear complexes (*R*,*R*,*R*,*R*)-**1** and (*R*,*R*,*S*,*S*)-**1**. When the catalyst loading was reduced to one fourth, the TOFs of the dinuclear complex (*R*,*R*,*R*,*R*)-**1** and the mononuclear complex (*R*,*R*)-**3** fell to about one half and one quarter, respectively (Table 1, entries 5 and 8). Thus, the dinuclear complex (*R*,*R*,*R*,*R*)-**1** was found to be less affected by the catalyst loading. It is unclear whether the copolymerization proceeds via bimetallic propagation mechanism in the present catalyst systems. Nevertheless, these results indicated that the linking of two (or more) cobalt–salen complexes is a promising design for highly active catalysts.

The effect of substituents on the salen moieties and the axial ligands on the cobalt centers was also investigated. As mentioned above, fluoro substituents at 5-positions of the salicylidene moieties and a nitrate axial ligand on the cobalt center were reported to be an optimal combination for the PO/MA copolymerization with mononuclear cobalt–salen complexes [15]. Therefore, we expected that the dinuclear cobalt–salen complexes (*R*,*R*,*R*,*R*)-**2** and (*R*,*R*,*S*,*S*)-**2** would give higher catalytic activities than complexes **1**. The homochiral and heterochiral complexes **2** efficiently copolymerized PO with PA and showed TOFs of 33 and 12 h^−1^, respectively, again demonstrating dependence of the catalytic activity on the absolute configuration (Table 1, entries 10 and 12). However, the TOFs unexpectedly are much lower than those obtained by using (*R*,*R*,*R*,*R*)-**1** and (*R*,*R*,*S*,*S*)-**1**. This trend was also observed for mononuclear complexes: the mononuclear complex *rac*-**4** with fluoro substituents at the 5-positions and a nitrate axial ligand gave a much lower TOF than *rac*-**3** (Table 1, entry 13). In addition, the TOF obtained with (*R*,*R*,*R*,*R*)-**2** and [PPN][OCOC_6_F_5_] was almost identical to that with [PPN][NO_3_] (Table 1, entries 11 and 10). Therefore, *tert*-butyl substituents and a pentafluorobenzoate axial ligand were found to be more suitable for the PO/PA copolymerization. It should also be noted that the linking of two complexes has a negative impact on catalytic activity in the case of the cobalt–salen complex with a fluoro substituent and a nitrate axial ligand (Table 1, entries 10 and 13).

### Monomer scope

**Scheme 2:**
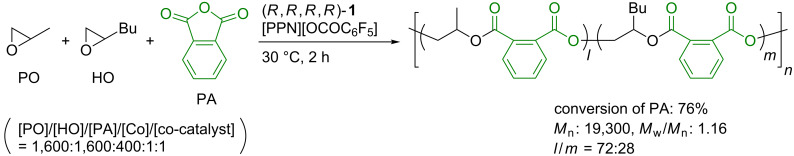
Terpolymerization of PO, HO, and PA with (*R*,*R*,*R*,*R*)-**1**.

In order to evaluate the copolymerization scope, the dinuclear cobalt–salen complex (*R*,*R*,*R*,*R*)-**1** and co-catalyst [PPN][OCOC_6_F_5_] were employed for the copolymerization of other epoxides with PA (Table 2). 1-Hexene oxide (HO), a terminal epoxide with an expanded alkyl chain, can be copolymerized at 30 °C to afford a completely alternating copolymer, while the TOF (61 h^−1^) was much lower than that obtained for the PO/PA copolymerization (Table 2, entry 1). A higher polymerization temperature improved the TOF up to 399 h^−1^ and almost complete conversion of PA was achieved within 1 h (Table 2, entry 2). The copolymerization with cyclohexene oxide (CHO), a common alicyclic epoxide in epoxide/CA copolymerization, also gave the corresponding alternating copolymer (Table 2, entries 3 and 4). The TOF of 244 h^−1^ was achieved at 60 °C, which was the highest one ever reported for a CHO/PA copolymerization. The copolymerization of PO with other CAs was also tested to evaluate the scope. Cyclohexane dicarboxylic anhydride (CHDA) was successfully converted into the corresponding polyester with a TOF of 53 h^−1^ (Table 2, entry 5). The copolymerization with maleic anhydride (MA), a common CA in the epoxide/CA copolymerization, took place (Table 2, entries 6 and 7). However, the TOF was low even at a higher temperature. The mononuclear cobalt–salen complex with fluoro substituents at 5-positions of the salicylidene moieties and a nitrate axial ligand on the cobalt center was reported to be most active for PO/MA copolymerization [15]. Thus, we also applied complex (*R*,*R*,*R*,*R*)-**2** with [PPN][NO_3_] as co-catalyst in the copolymerization. As a result, the complex was found to show a slightly higher activity compared with (*R*,*R*,*R*,*R*)-**1** although the TOF was much lower than that obtained with the mononuclear complex (Table 2, entry 8). Finally, we attempted the terpolymerization of PO, HO, and PA (Scheme 2). Although equimolar amounts of PO and HO were used, the obtained copolymer contained a larger amount of the PO-derived repeating unit, reflecting a higher reactivity of PO.

**Table 2:** Copolymerization of various epoxides and cyclic anhydrides.^a^

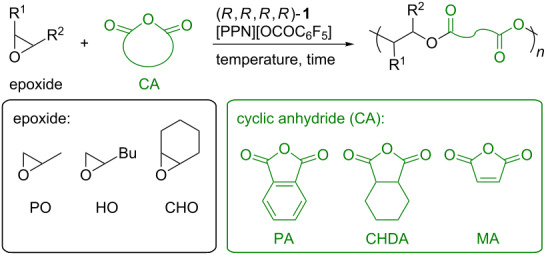							

entry	monomer	*T* (°C)	time (h)	[epoxide]/[CA]/[Co]/[co-catalyst]	TOF^b^ (h^−1^)	*M*_n_^c^	*M*_w_/*M*_n_^c^

1	HO/PA	30	2	2,400:400:1:1	61	8,400	1.20
2	HO/PA	60	1	2,400:400:1:1	399	22,000	1.18
3	CHO/PA	30	2	2,800:400:1:1	20	500	1.97
4	CHO/PA	60	1	2,800:400:1:1	244	7,000	1.16
5	PO/CHDA	30	2	4,000:400:1:1	53	1,400	2.06
6	PO/MA	30	2	2,000:200:1:1	10	–^d^	–^d^
7	PO/MA	60	1.5	2,000:200:1:1	39	–^d^	–^d^
8^e^	PO/MA	30	2	2,000:200:1:1	22	–^d^	–^d^

^a^Copolymerization conditions: epoxide, CA (2.0 mmol for entries 1–5; 1.0 mmol for entries 6–8), cobalt complex, [PPN][OCOC_6_F_5_] as a co-catalyst. ^b^Turnover frequency (TOF) = (mol of CA incorporated in the copolymer)∙(mol of Co center)^−1^∙h^−1^ calculated based on the ^1^H NMR spectrum of the polymerization mixture using phenanthrene as an internal standard. ^c^Estimated by size-exclusion-chromatography analysis using a polystyrene standard. ^d^Not determined because of low conversion of MA. ^e^(*R*,*R*,*R*,*R*)-**2** and [PPN][NO_3_].

## Conclusion

We reported the alternating copolymerization of epoxides with cyclic anhydrides using dinuclear cobalt–salen complexes with a benzene linker. The substituents on the salen moieties, an axial ligand on the cobalt center, and a combination of the absolute configuration of the two cobalt–salen moieties were found to have a great impact on the catalytic activity. Through these investigations, the homochiral dinuclear cobalt–salen complex having *tert*-butyl substituents and a pentafluorobenzoate axial ligand was developed as a highly active catalyst. Furthermore, the dinuclear cobalt–salen complex demonstrated a higher catalytic activity than the corresponding mononuclear cobalt–salen complex. These results indicate that the design based on di- or multinuclear metal complexes is a promising strategy for the development of highly active catalysts. Our future studies will focus on the optimization of linker structures as well as ligand structures for the epoxide/CA copolymerization to achieve higher activity and/or selectivity.

## Experimental

**General procedures:** All manipulations involving air and/or moisture-sensitive compounds were carried out using standard Schlenk techniques under argon. Analytical thin-layer chromatography was performed on glass plates coated with 0.25 mm 230–400 mesh silica gel containing a fluorescence indicator. Column chromatography was performed on silica gel (spherical neutral, particle size: 63–210 μm). Most of the reagents were purchased from commercial suppliers, such as Sigma-Aldrich Co. LLC, Tokyo Chemical Industry Co., Ltd., and Kanto Chemical Co., Inc., and were used without further purification unless otherwise specified. Commercially available anhydrous solvents were used for air and/or moisture-sensitive reactions. Epoxides for the polymerizations were dried over CaH_2_ and distilled under argon and cyclic anhydrides were purified by sublimation. (*R*,*R*,*S*,*S*)- and (*R*,*R*,*R*,*R*)-**1** [40], (*R*,*R*)- and *rac*-**3** [42], *rac*-**4** [15], **5** [40], [PPN][OCOC_6_F_5_] [42], and [PPN][NO_3_] [15] were prepared according to the literature.

NMR spectra were recorded in CDCl_3_ on a JEOL–ECX400 spectrometer (^1^H NMR at 400 MHz; ^13^C NMR at 101 MHz) or a JEOL–ECA500 spectrometer (^19^F NMR at 471 MHz). Chemical shifts are reported in ppm relative to the internal standard signal (0 ppm for Me_4_Si in CDCl_3_) for ^1^H NMR and the solvent signal (77.16 ppm for CDCl_3_) for ^13^C NMR. Data are presented as follows: chemical shift, multiplicity (s = singlet, d = doublet, dd = doublet of doublets, m = multiplet and/or multiple resonances), coupling constant in hertz (Hz), and signal area integration in natural numbers. High resolution mass spectra are taken with a Bruker Daltonics micrOTOF-QII mass spectrometer by atmospheric pressure chemical ionization–time-of-flight (APCI–TOF) method. Size-exclusion-chromatography (SEC) analyses for evaluating molecular weights were carried with two columns (Shodex KF-804L) using chloroform as an eluent at 40 °C at 1 mL/min. The molecular weight was calibrated against standard polystyrene samples. The recycling preparative HPLC was performed with YMC-GPC T-2000 and T-4000 columns (chloroform as an eluent). HPLC analysis for determining the ee was carried out using a DAICEL CHIRALCEL^®^ IA-3 column (4.6 mm × 250 mm).

**Synthesis of mono(salen) (*R*,*R*)-7:** A Schlenk tube was charged with (*R,R*)-1,2-cyclohexanediamine monohydrochloride (0.14 g, 0.93 mmol), 3-*tert*-butyl-5-fluoro-2-hydroxybenzaldehyde (0.18 g, 0.93 mmol), molecular sieves 4Å, and dry MeOH (1.5 mL) under argon. After stirring at room temperature for 50 min, the resulting solution was slowly transferred to another Schlenk tube containing bis(salicylaldehyde) **5** (0.72 g, 1.4 mmol), Et_3_N (0.47 mL, 3.4 mmol) and dichloromethane (7 mL). The reaction mixture was stirred at room temperature for 18 h and the resulting suspension was filtered off with dichloromethane. The filtrate was concentrated under reduced pressure and the crude residue was purified by silica-gel column chromatography (AcOEt/hexane/Et_3_N 1:5:0.12 as an eluent, *R*_f_ 0.30] to provide (*R*,*R*)-**7** (0.36 g, 39% yield) as yellow solid: ^1^H NMR (400 MHz, CDCl_3_) δ 11.73 (s, 1H), 9.75 (s, 1H), 8.24 (s, 1H), 8.19 (s, 1H), 7.99–7.92 (m, 2H), 7.71–7.67 (m, 2H), 7.29 (s, 2H), 7.04 (d, *J* = 2.7 Hz, 1H), 6.98 (dd, *J* = 11.0, 3.2 Hz, 1H), 6.90 (d, *J* = 2.7 Hz, 1H), 6.70 (dd, *J* = 7.8, 3.2Hz, 1H), 3.40–3.28 (m, 2H), 2.00–1.88 (m, 4H), 1.76–1.68 (m, 2H), 1.52–1.45 (m, 2H), 1.36 (s, 9H), 1.32 (s, 18H); ^13^C NMR (101 MHz, CDCl_3_) δ 196.5, 166.5, 166.3, 164.7, 159.3, 158.6, 156.5, 155.0 (d, *J*_C–F_ = 234.8 Hz), 142.6, 141.7, 140.4, 139.4 (d, *J*_C–F_ = 4.8 Hz), 139.1, 132.0, 131.9, 131.8, 131.7, 129.8, 129.6, 128.1, 128.0, 123.4, 122.9, 121.3, 120.2, 118.3, 118.1 (d, *J*_C–F_ = 7.7 Hz), 117.1 (d, *J*_C–F_ = 24.9 Hz), 114.2 (d, *J*_C–F_ = 22.0 Hz), 72.7, 72.1, 35.1, 35.0, 33.2, 33.0, 29.23, 29.15, 29.0, 24.34, 24.29; ^19^F NMR (471 MHz, CDCl_3_) δ −126.3; HRMS–APCI^+^ (*m*/*z*): [M + H]^+^ calcd for C_47_H_54_FN_2_O_8_, 793.3859; found, 793.3859.

**Synthesis of bis(salen) (*R,R*,*S,S*)-8:** The crude product was obtained from (*S,S*)-1,2-cyclohexanediamine monohydrochloride (28 mg, 0.19 mmol), 3-*tert*-butyl-5-fluoro-2-hydroxybenzaldehyde (37 mg, 0.19 mmol), (*R*,*R*)-**7** (0.13 g, 0.17 mmol), and Et_3_N (0.11 mL, 0.79 mmol) according to the procedure described for the synthesis of (*R*,*R*)-**7**. Purification by silica gel column chromatography (AcOEt/hexane/Et_3_N 1:5:0.12 as an eluent, *R*_f_ 0.38) gave (*R,R*,*S,S*)-**8** (95 mg, 52% yield) as yellow solid: ^1^H NMR (400 MHz, CDCl_3_) δ 8.24 (s, 2H), 8.17 (s, 2H), 7.93–7.89 (m, 2H), 7.67–7.62 (m, 2H), 7.01 (d, *J* = 2.7 Hz, 2H), 6.98 (dd, *J* = 11.0, 3.2 Hz, 1H), 6.88 (d, *J* = 2.7 Hz, 2H), 6.70 (dd, *J* = 7.8, 2.7 Hz, 2H), 3.38–3.28 (m, 4H), 2.00–1.88 (m, 8H), 1.79–1.66 (m, 4H), 1.52–1.45 (m, 4H), 1.37 (s, 9H), 1.36 (s, 9H), 1.27 (s, 18H); ^13^C NMR (101 MHz, CDCl_3_) δ 166.4, 164.8, 164.7 (d, *J*_C–F_ = 1.9 Hz), 158.5, 156.5, 155.0 (d, *J*_C–F_ = 234.8 Hz), 141.8, 139.4 (d, *J*_C–F_ = 5.8 Hz), 139.0, 131.9, 131.8, 129.6, 123.0, 121.4, 118.3, 118.1 (d, *J*_C–F_ = 7.7 Hz), 117.1 (d, *J*_C–F_ = 24.0 Hz), 114.2 (d, *J*_C–F_ = 22.0 Hz), 72.8, 72.0, 35.1, 35.0, 33.2, 33.0, 29.3, 29.1, 24.35, 24.29; ^19^F NMR (471 MHz, CDCl_3_) δ −126.3; HRMS–APCI^+^ (*m*/*z*): [M + H]^+^ calcd for C_64_H_77_F_2_N_4_O_8_^+^, 1067.5704; found, 1067.5697.

**Synthesis of bis(salen) (*R,R*,*R,R*)-8:** The crude product was obtained from (*R*,*R*)-1,2-cyclohexanediamine monohydrochloride (0.10 g, 0.66 mmol), 3-*tert*-butyl-5-fluoro-2-hydroxybenzaldehyde (0.13 g, 0.66 mmol), bis(salicylaldehyde) **5** (0.15 g, 0.30 mmol), and Et_3_N (0.14 mL, 1.0 mmol) according to the procedure described for the synthesis of (*R*,*R*)-**7**. Purification by silica gel column chromatography (AcOEt/hexane/Et_3_N 1:5:0.12 as an eluent, *R*_f_ 0.38) gave (*R,R*,*R,R*)-**8** (145 mg, 45% yield) as yellow solid: ^1^H NMR (400 MHz, CDCl_3_) δ 8.24 (s, 2H), 8.17 (s, 2H), 7.93–7.89 (m, 2H), 7.68–7.63 (m, 2H), 7.01 (d, *J* = 3,2 Hz, 2H), 6.98 (dd, *J* = 11.0, 2.7 Hz, 1H), 6.88 (d, *J* = 2.7 Hz, 2H), 6.70 (dd, *J* = 7.8, 3.2 Hz, 2H), 3.38–3.27 (m, 4H), 2.00–1.88 (m, 8H), 1.79–1.66 (m, 4H), 1.52–1.44 (m, 4H), 1.36 (s, 18H), 1.27 (s, 18H); ^13^C NMR (101 MHz, CDCl_3_) δ 166.5, 164.8, 164.7 (d, *J*_C–F_ = 2.9 Hz), 158.5, 156.5, 155.0 (d, *J*_C–F_ = 234.8 Hz), 141.8, 139.4 (d, *J*_C–F_ = 5.8 Hz), 139.0, 131.9, 131.8, 129.6, 123.0, 121.4, 118.3, 118.1 (d, *J*_C–F_ = 7.7 Hz), 117.1 (d, *J*_C–F_ = 24.0 Hz), 114.2 (d, *J*_C–F_ = 23.0 Hz), 72.8, 72.0, 35.1, 35.0, 33.2, 33.0, 29.2, 29.1, 24.34, 24.28; HRMS–APCI^+^ (*m*/*z*): [M + H]^+^ calcd for C_64_H_77_F_2_N_4_O_8_^+^, 1067.5704; found, 1067.5702.

**Synthesis of dinuclear Co–salen complex (*R*,*R*,*S*,*S*)-2:** A Schlenk tube was charged with Co(NO_3_)_2_·6H_2_O (52 mg, 0.18 mmol). After stirring at 60 °C under reduced pressure for 1.5 h, EtOH (2.0 mL) was added. Another Schlenk tube was charged with (*R,R*,*S,S*)-**8** (88 mg, 80 μmol) and degassed CH_2_Cl_2_ (3.0 mL) added under argon. After the solution of Co(NO_3_)_2_ was slowly added to the ligand solution, the resulting mixture was stirred at room temperature for 1 h, and then opened to air. The reaction mixture was stirred at room temperature for 13 h, filtered, and concentrated under reduced pressure. The resulting residue was rinsed with pentane until the filtrate was clear, and then dried at 60 °C under reduced pressure to provide (*R,R*,*S,S*)-**2** (94 mg, 90% yield for two steps) as dark green powder: HRMS–APCI^+^ (*m*/*z*): [M + H – 2(NO_3_)]^+^ calcd for C_64_H_73_Co_2_F_2_N_4_O_8_^+^, 1181.4055; found, 1181.4054; anal. calcd for C_64_H_72_Co_2_F_2_N_6_O_14_ (%): C, 58.90; H, 5.56; N, 6.44; found: C, 55.84; H, 5.82; N, 5.33.

**Synthesis of dinuclear Co–salen complex (*R,R*,*R,R*)-2:** Complex (*R,R*,*R*,*R*)-**2** was obtained from Co(NO_3_)_2_·6H_2_O (35 mg, 0.12 mmol), (*R,R*,*R,R*)-**8** (58 mg, 54 μmol) as dark green powder (62 mg, 88% yield for two steps) according to the procedure described for the synthesis of (*R*,*R*,*S*,*S*)-**2**: HRMS–APCI^+^ (*m*/*z*): [M + H – 2(NO_3_)]^+^ calcd for C_64_H_73_Co_2_F_2_N_4_O_8_^+^, 1181.4055; found, 1181.4055; anal. calcd for C_64_H_72_Co_2_F_2_N_6_O_14_ (%): C, 58.90; H, 5.56; N, 6.44; found: C, 59.23; H, 5.93; N, 5.06.

**Representative procedure for the copolymerization of propylene oxide with phthalic anhydride (**Table 1**):** A flame-dried Schlenk tube was charged with the propylene oxide (1.4 mL, 20 mmol), phthalic anhydride (296 mg, 2.0 mmol), cobalt complex (5.0 μmol of Co center), and co-catalyst (5.0 μmol [PO]/[PA]/[Co]/[co-catalyst] = 4,000:400:1:1) under argon. The reaction mixture was stirred at 30 °C for 1.0 h. The polymerization mixture was diluted with CH_2_Cl_2_, and quenched with several drops of MeOH/1 M HCl (50:50 vol %). Phenanthrene as an internal standard was dissolved in the resulting mixture, and a small aliquot of the mixture was taken out and concentrated under reduced pressure. Then, the residue was analyzed by ^1^H NMR spectroscopy and SEC to determine the conversion of PA, TOF, molecular weight, and molecular weight distribution.

The ^1^H NMR spectra of the obtained PO/PA [15], CHO/PA [33], PO/CHDA [21], and PO/MA [15] copolymers were identical to those in the literatures.

**Evaluation of regioregularity:** The copolymerization of (*S*)-PO and PA was carried out under standard conditions using (*R*,*R*,*R*,*R*)-**1**. The reaction mixture was diluted with CH_2_Cl_2_ and quenched with several drops of MeOH/1 M HCl (50:50 vol %). The resulting mixture was poured into an excess of MeOH to precipitate the copolymer. After the precipitation was repeated two more times, the precipitate was collected and dried under deduced pressure.

The obtained copolymer (ca. 150 mg) was dissolved in a mixture of CH_2_Cl_2_ (2.5 mL) and MeOH (7.5 mL), and NaOH (100 mg) was added. After stirring at 60 °C for 25 h, the resulting suspension was neutralized with 4 M HCl (in cyclopentyl methyl ether). The solvents were removed under reduced pressure and Et_2_O (5 mL) was added to the resulting powder. After stirring for 10 min, the slurry was filtrated off and the resulting filtrate was concentrated under reduced pressure to give propylene glycol.

A flame-dried flask was charged with the obtained propylene glycol (26 mg, 0.34 mmol), 4-(dimethylamino)pyridine (8 mg, 0.07 mmol), trimethylamine (70 µL, 0.7 mmol), and CH_2_Cl_2_ (3.4 mL). Benzoyl chloride (39 µL, 0.34 mmol) was added dropwise to the solution with stirring. After stirring at room temperature for 25 min, the reaction mixture was poured into water and extracted with CH_2_Cl_2_. The organic layer was washed with 1 M HCl and brine and concentrated under reduced pressure. The resulting crude product was purified by the recycling preparative HPLC to give 2-hydroxypropyl benzoate [43]. The ee of the benzoate was determined by HPLC analysis using a DAICEL CHIRALCEL^®^ IA-3 column [*t*_R_ = 12.65 min for the (*S*)-isomer and 14.50 min for the (*R*)-isomer (flow rate: 1.0 mL; eluent: hexane/iPrOH 95:5)].

## Supporting Information

File 1 NMR spectra of new compounds.
