# Supplementary material for: Copolymerization of epoxides with cyclic anhydrides catalyzed by dinuclear cobalt complexes
Source: Beilstein J Org Chem. 2018 Nov 5;14:2779–88. doi: 10.3762/bjoc.14.255 (PMC6244378; doi:10.3762/bjoc.14.255)
Supplement: File 1 — NMR spectra of new compounds. [file Beilstein_J_Org_Chem-14-2779-s001.pdf]

Supporting Information

for

Copolymerization of epoxides with cyclic anhydrides catalyzed by dinuclear cobalt complexes

Yo Hiranoi and Koji Nakano*

Address: Department of Organic and Polymer Materials Chemistry, Tokyo University of Agriculture and Technology, 2-24-16 Naka-cho, Koganei, Tokyo 184-8588, Japan

Email: Koji Nakano - k_nakano@cc.tuat.ac.jp

* Corresponding author

NMR spectra of new compounds

Table of Contents

^{1}H, ^{13}C, and ^{19}F NMR spectra of (*R*,*R*)-**7** S2

^{1}H, ^{13}C, and ^{19}F NMR spectra of (*R*,*R*,*S*,*S*)-**8** S4

^{1}H, ^{13}C, and ^{19}F NMR spectra of (*R*,*R*,*R*,*R*)-**8** S6

^{1}H and ^{13}C NMR spectra of the HO/PA copolymer S8

^{1}H and ^{13}C NMR spectra of the PO/HO/PA terpolymer S9

MALDI–TOF mass spectrum of the PO/PA copolymer S10

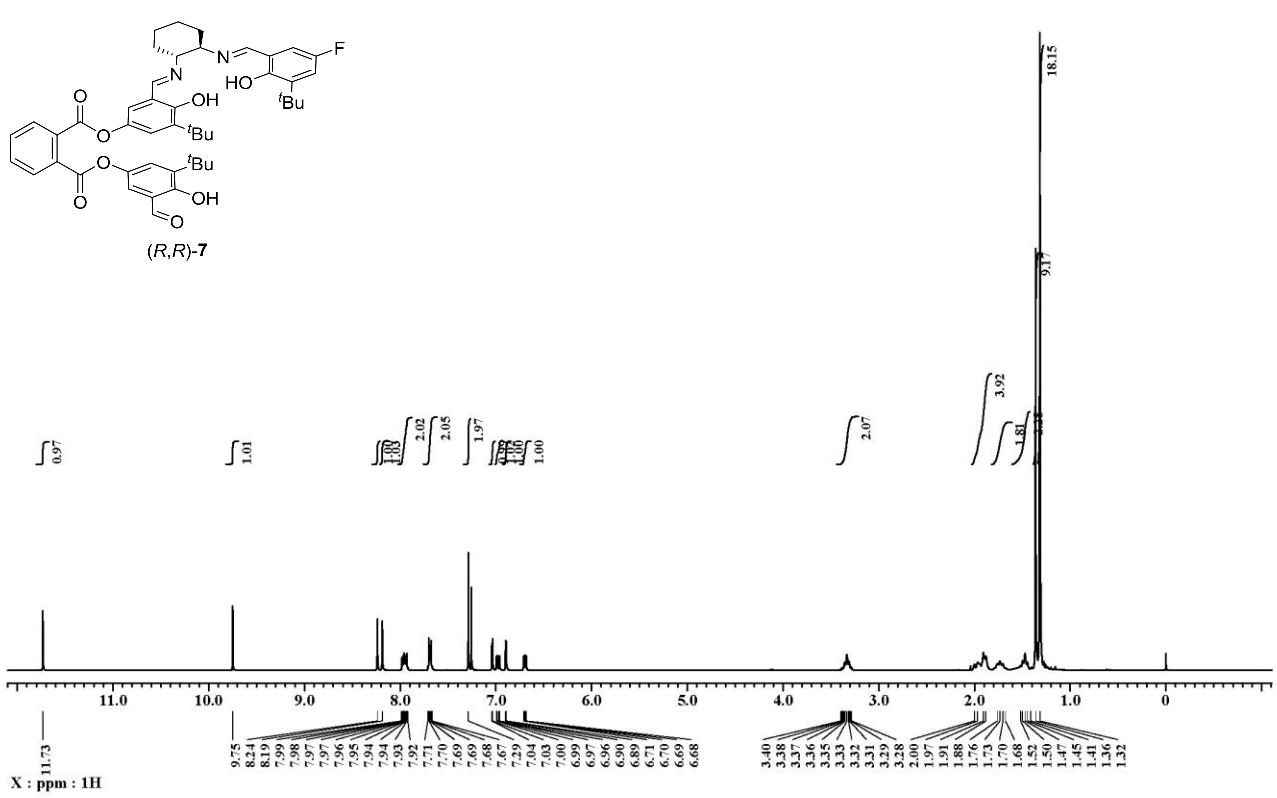

Figure S1: ^{1}H NMR spectrum of (*R*,*R*)-**7** (400 MHz, $CDCl_3$).

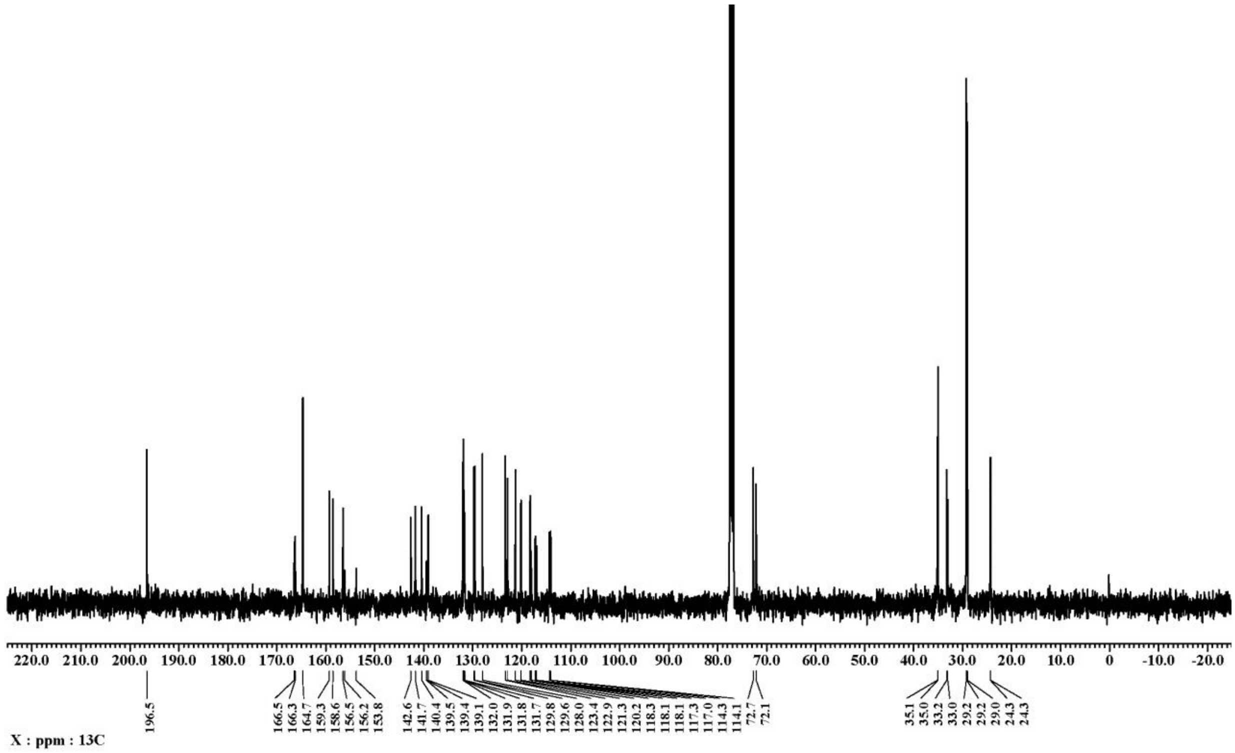

Figure S2: ^{13}C NMR spectrum of (*R*,*R*)-**7** (101 MHz, $CDCl_3$).

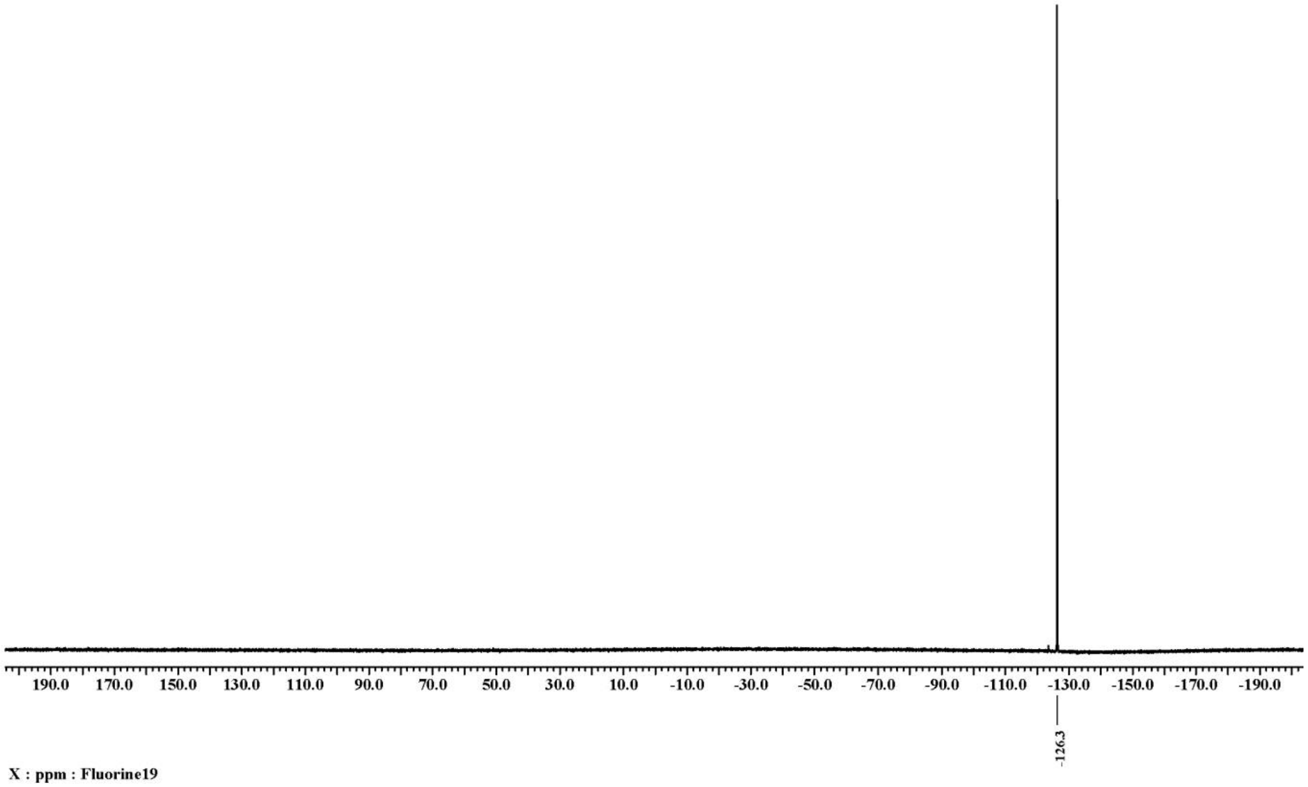

Figure S3: ^{19}F NMR spectrum of (*R*,*R*)-**7** (471 MHz, $CDCl_3$).

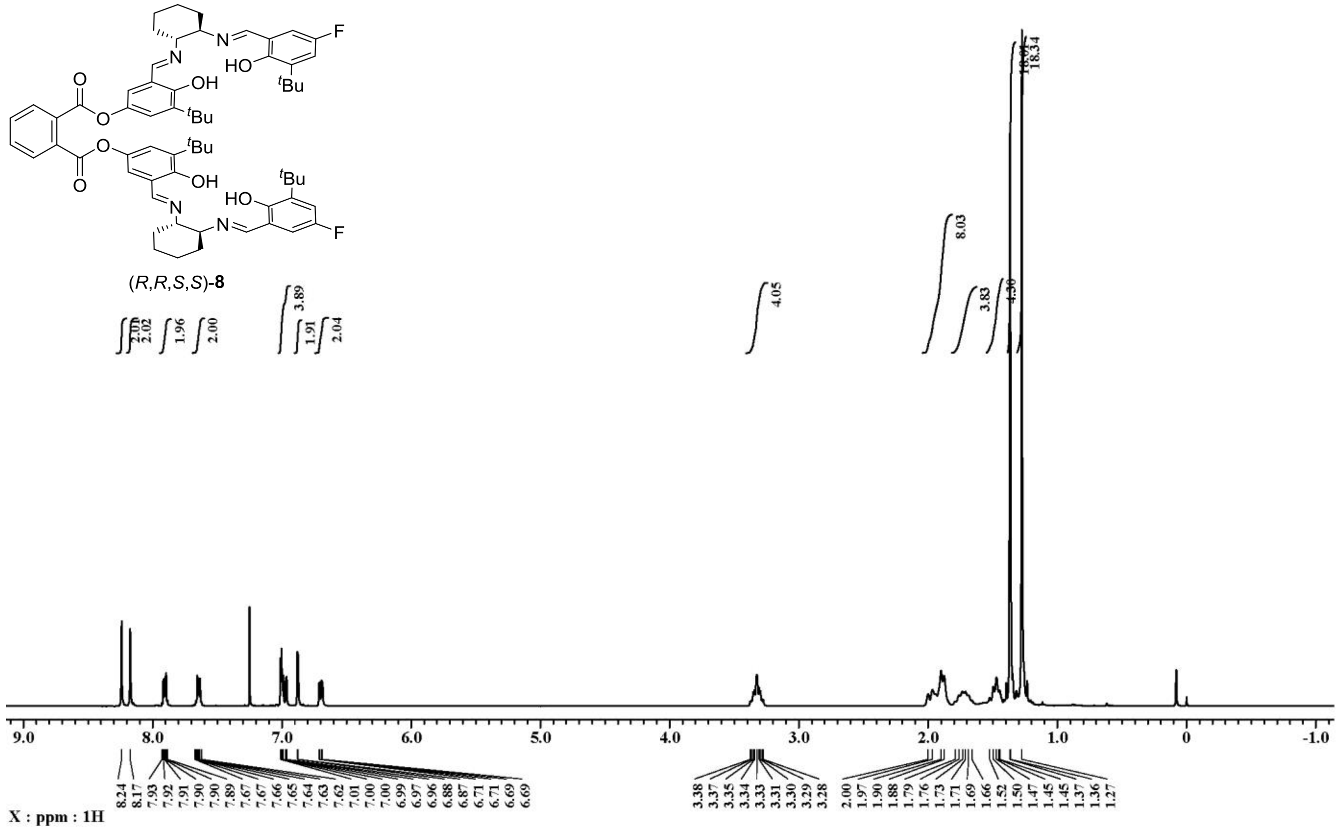

Figure S4: ^{1}H NMR spectrum of (*R*,*R*,*S*,*S*)-**8** (400 MHz, $CDCl_3$).

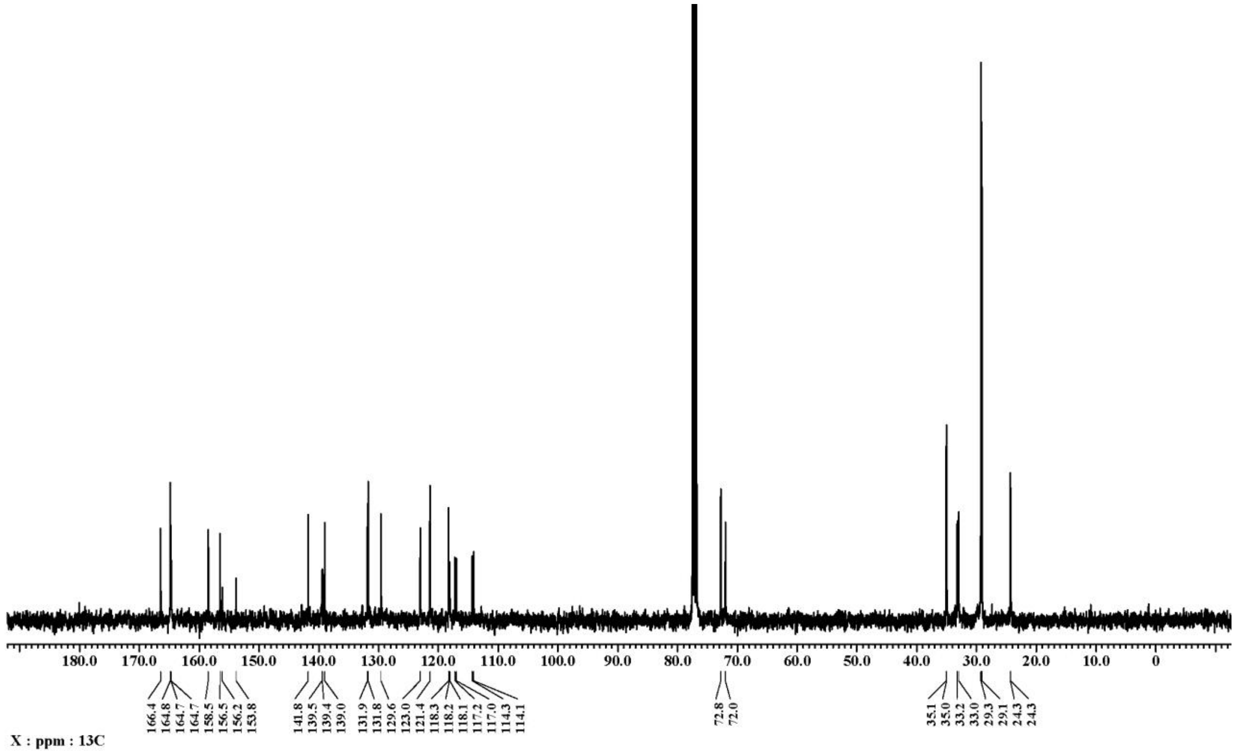

Figure S5: ^{13}C NMR spectrum of (*R*,*R*,*S*,*S*)-**8** (101 MHz, $CDCl_3$).

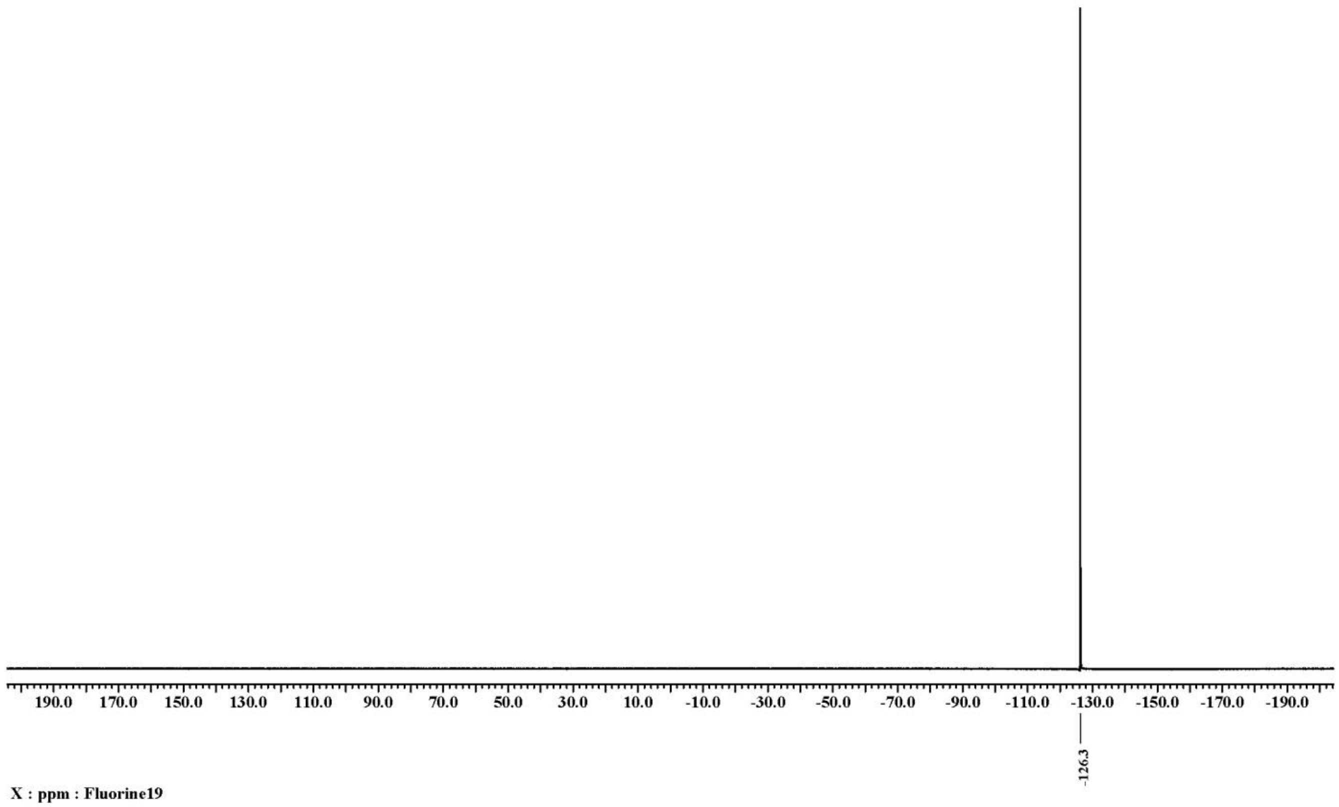

Figure S6: ^{19}F NMR spectrum of (*R*,*R*,*S*,*S*)-**8** (471 MHz, $CDCl_3$).

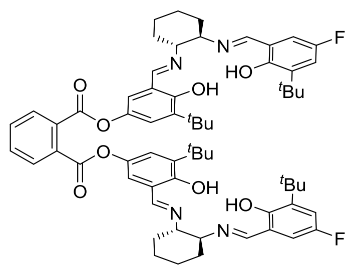

(*R*,*R*,*R*,*R*)-**8**

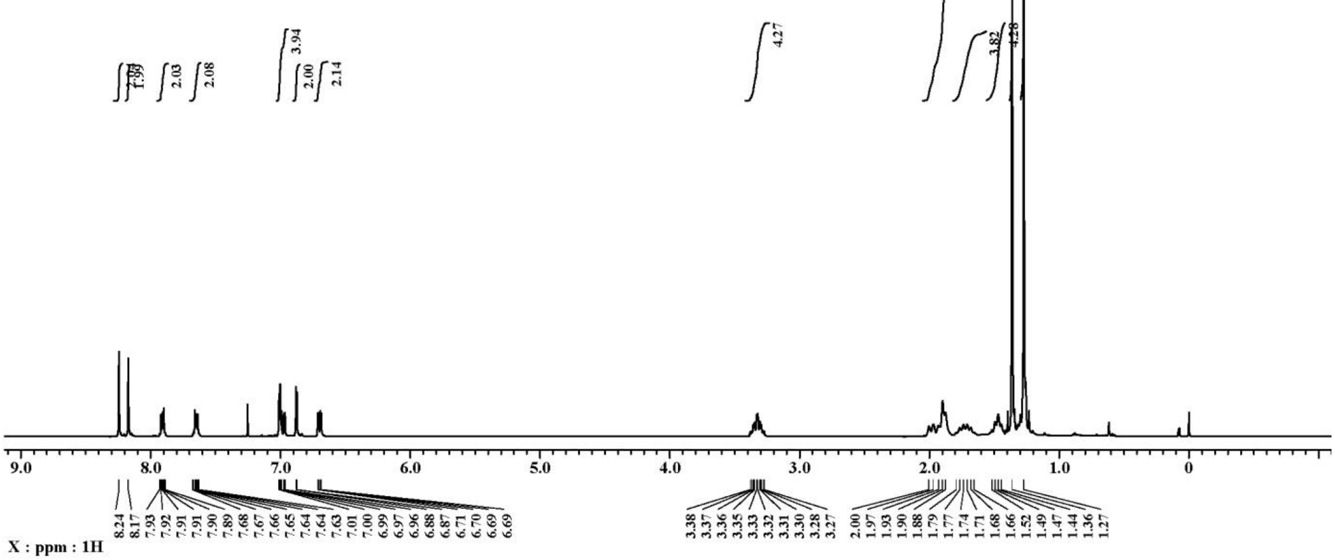

Figure S7: ^{1}H NMR spectrum of (*R*,*R*,*R*,*R*)-**8** (400 MHz, $CDCl_3$).

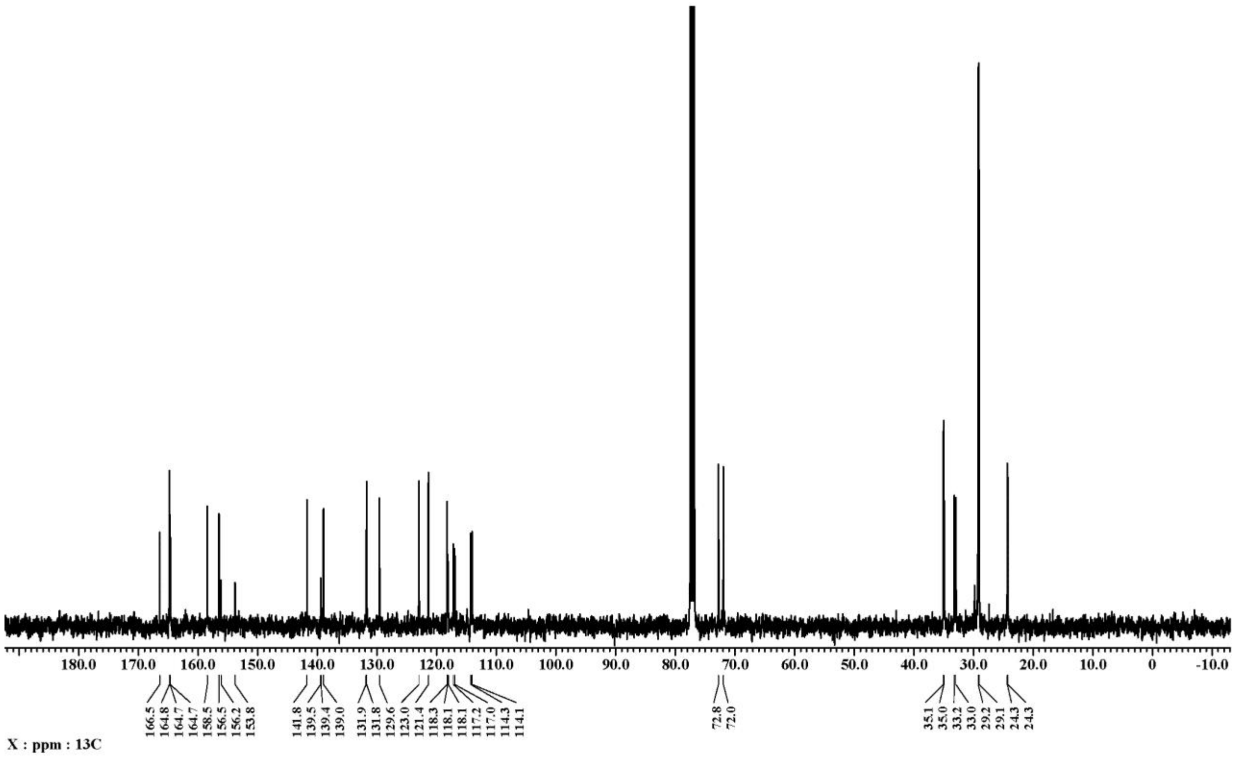

Figure S8: ^{13}C NMR spectrum of (*R*,*R*,*R*,*R*)-**8** (101 MHz, $CDCl_3$).

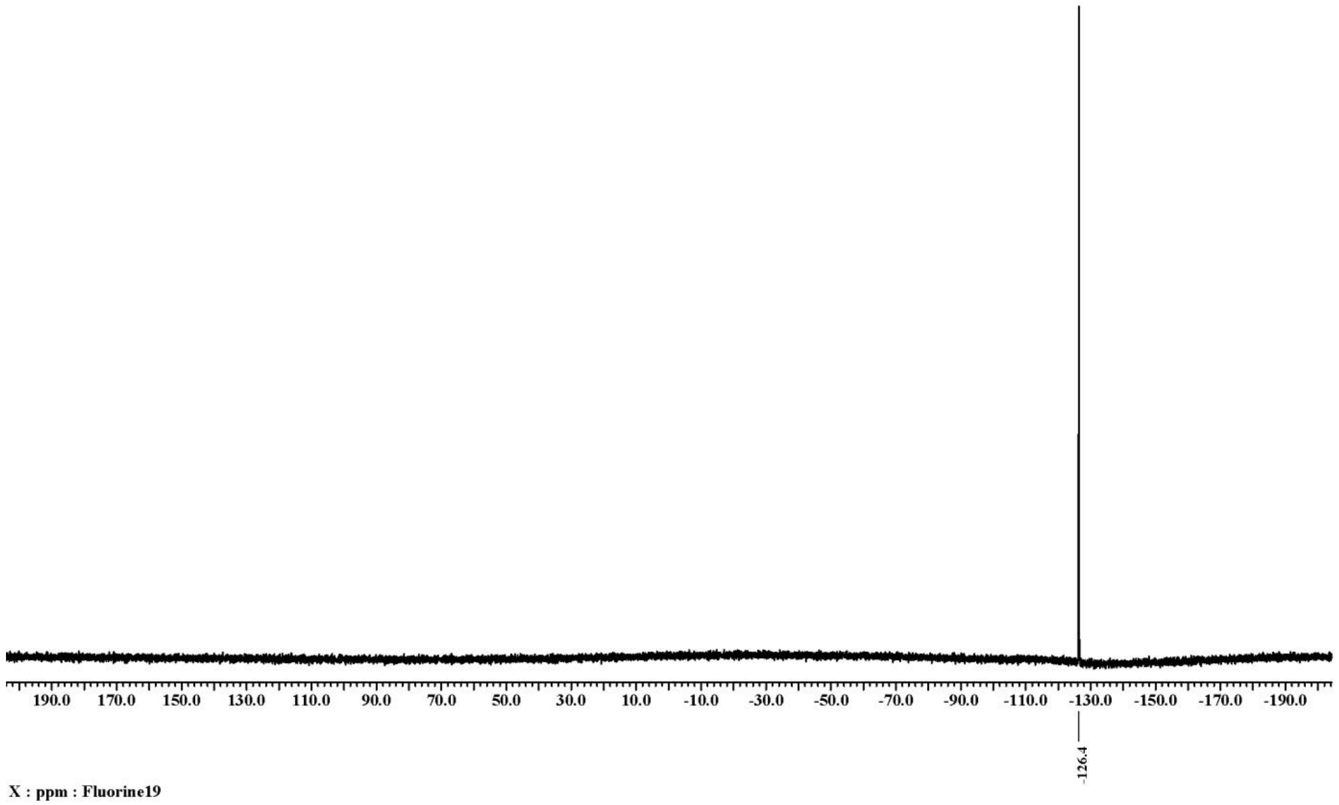

Figure S9: ^{19}F NMR spectrum of (*R*,*R*,*R*,*R*)-**8** (471 MHz, $CDCl_3$).

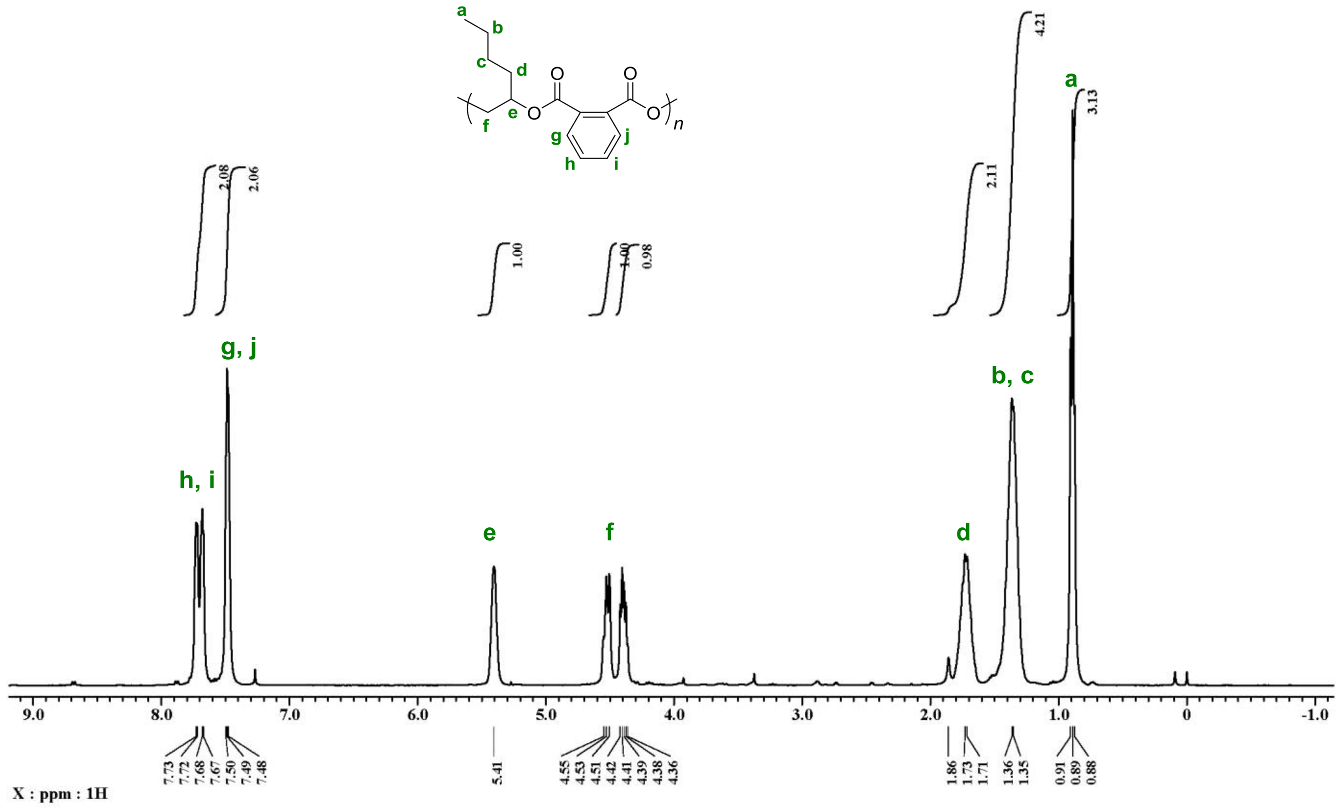

Figure S10: ^{1}H NMR spectrum of HO/PA copolymer (400 MHz, $CDCl_3$).

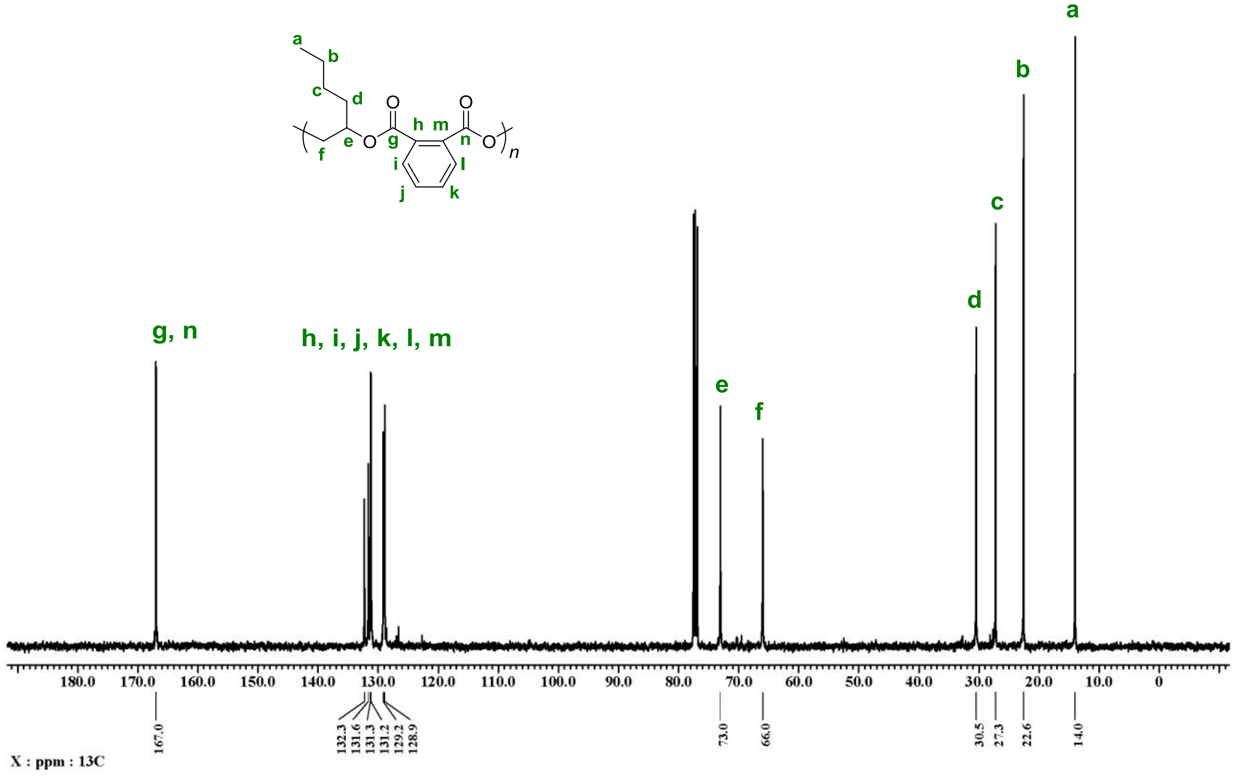

Figure S11: ^{13}C NMR spectrum of HO/PA copolymer (101 MHz, $CDCl_3$).

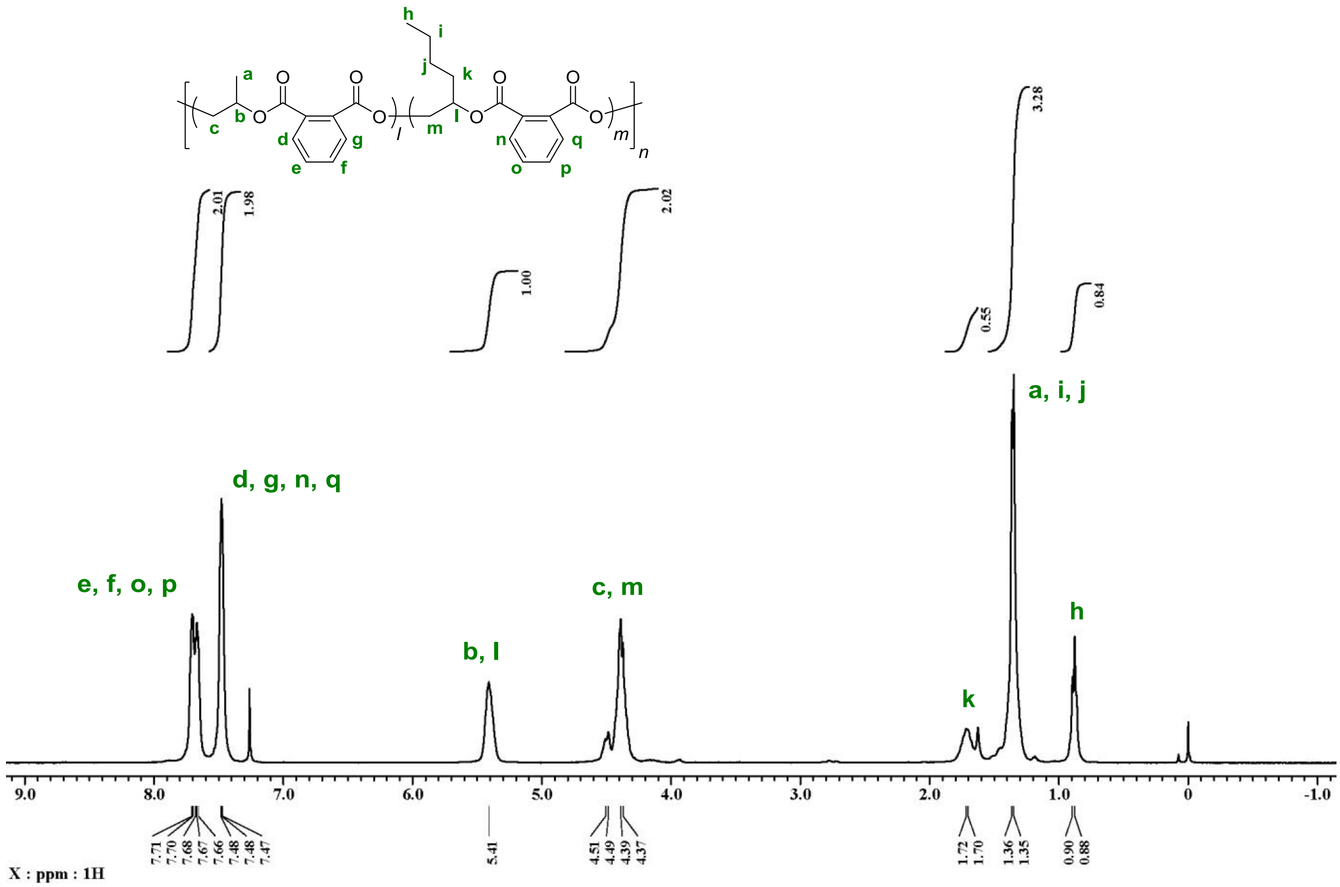

Figure S12: ^{1}H NMR spectrum of PO/HO/PA terpolymer (400 MHz, $CDCl_3$).

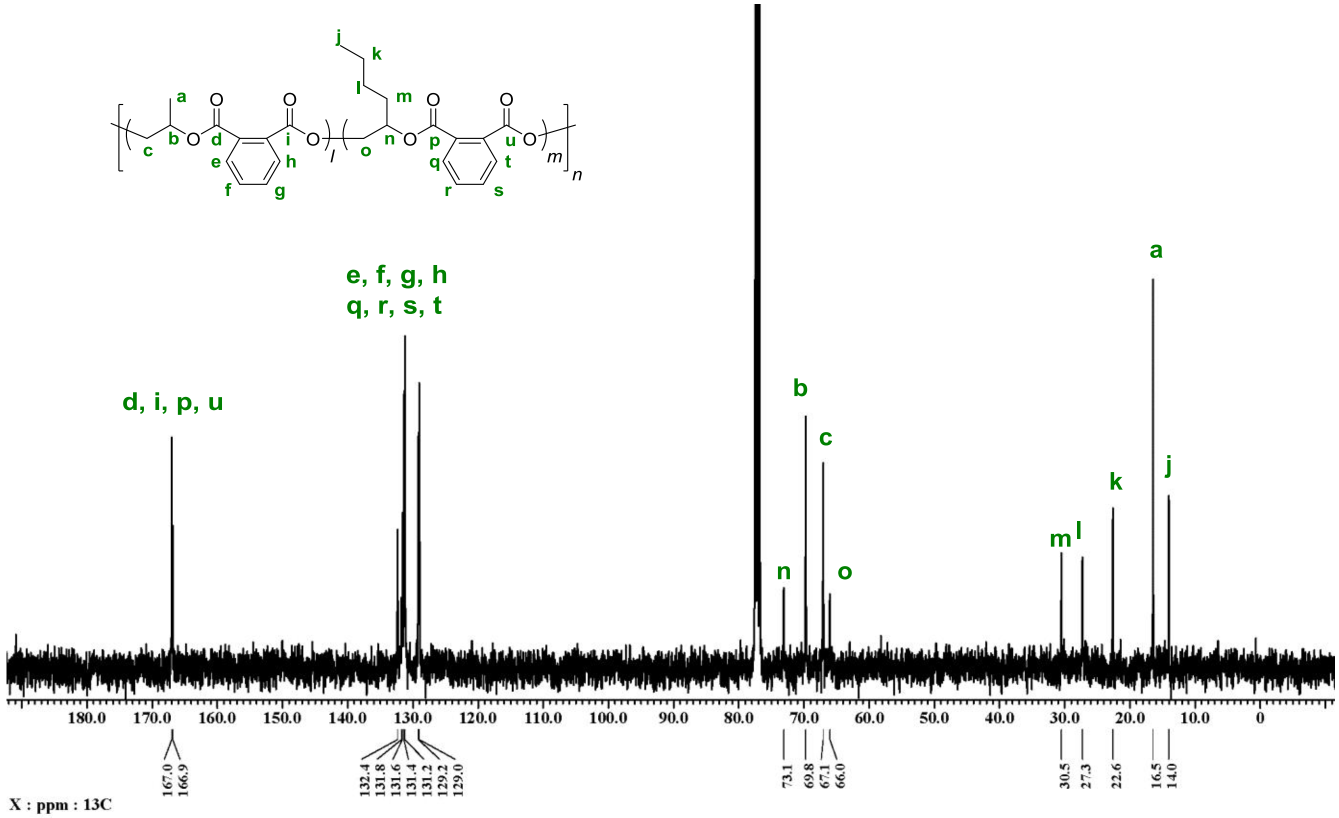

Figure S13: ^{13}C NMR spectrum of PO/HO/PA terpolymer (101 MHz, $CDCl_3$).

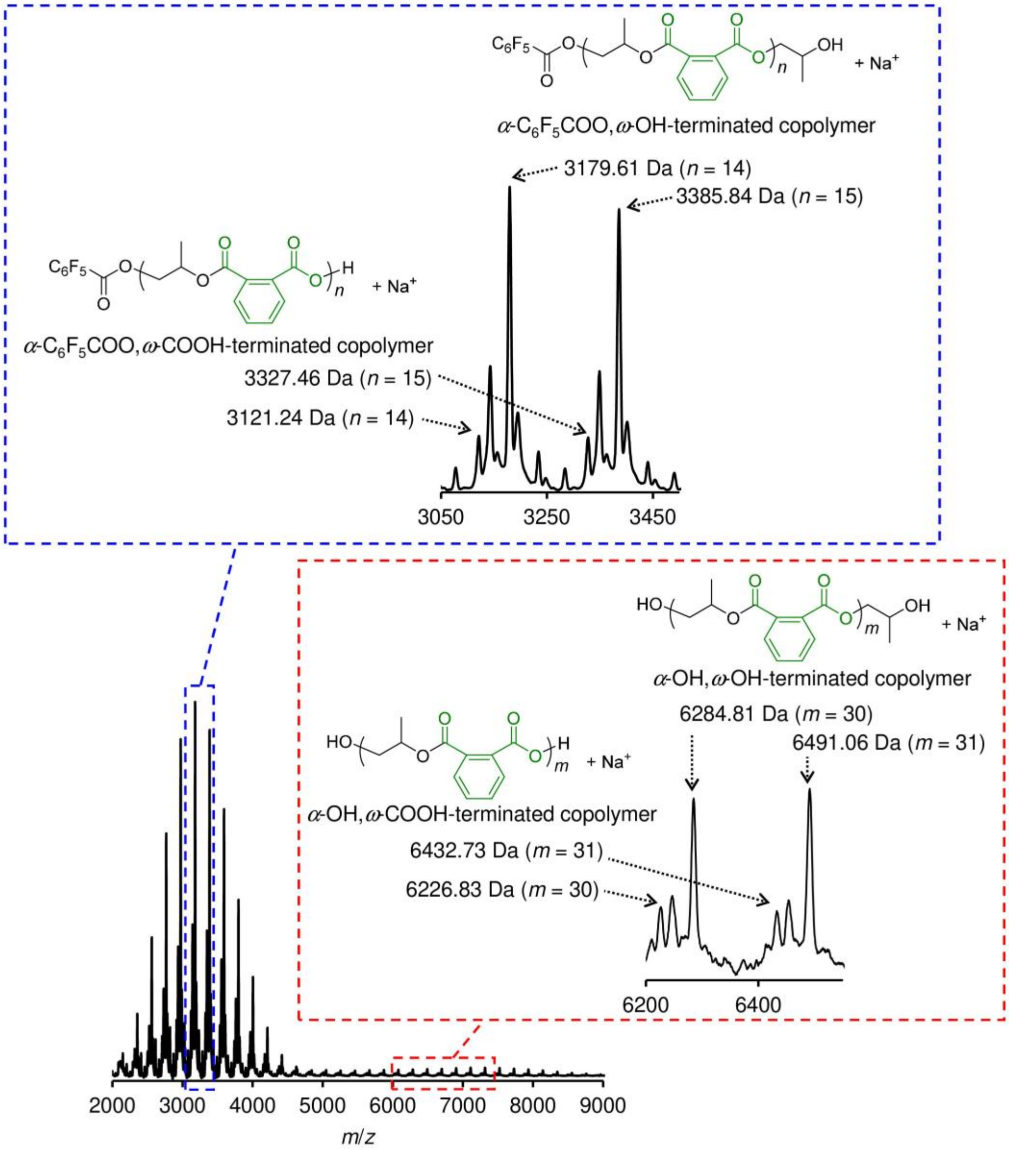

Figure S14: MALDI–TOF mass spectrum of the PO/PA copolymer (the low-molecular weight copolymer for MS analysis was prepared by using (*R*,*R*,*R*,*R*)-**1** at 30 °C for 15 min).
